# Effects of paraprobiotic *Lactobacillus acidophilus* LA‐5 on PANoptosis, oxidative stress and inflammation in acetic acid‐induced ulcerative colitis

**DOI:** 10.1002/jsfa.71037

**Published:** 2026-09-04

**Authors:** Adem Yavaş, Ezgi Özkıran, Ecem Akan

**Affiliations:** ^1^ Food Quality Control and Analysis, Food Processing Department, Çine Vocational School Aydın Adnan Menderes University Aydın Turkey; ^2^ Department of Pathology, Faculty of Medicine Aydın Adnan Menderes University Aydın Turkey; ^3^ Department of Dairy Technology, Faculty of Agriculture Aydın Adnan Menderes University Aydın Turkey

**Keywords:** ulcerative colitis, paraprobiotic, *lactobacillus acidophilus* LA‐5, PANoptosis, ZBP1, short‐chain fatty acids

## Abstract

**BACKGROUND:**

Ulcerative colitis (UC) is a relapsing inflammatory bowel disease characterised by oxidative stress, inflammation and regulated cell death of the colonic mucosa. Because live probiotics may cause bacteraemia and sepsis in immunocompromised hosts, non‐viable (paraprobiotic) alternatives have attracted interest.

**METHODS:**

This study evaluated the antioxidant, anti‐inflammatory and PANoptosis‐regulating effects of a heat‐inactivated paraprobiotic derived from *Lactobacillus acidophilus* LA‐5 in acetic acid (AA)‐induced experimental UC, compared to the live probiotic. Forty female Balb/c mice were assigned to four groups (*n* = 10): healthy control, AA colitis, live LA‐5 (AA‐PRO) and heat‐inactivated paraprobiotic (AA‐PARA). Colitis was induced by intrarectal administration of 4% acetic acid, and treatments were given by oral gavage for 7 days. Disease activity, colon histopathology, oxidant/antioxidant biomarkers, serum cytokines, PANoptosis‐related proteins (western blot) and caecal short‐chain fatty acids were assessed.

**RESULTS:**

AA increased the disease activity index, shortened the colon and induced splenomegaly, histopathological damage, oxidative stress and a systemic cytokine surge. Both treatments limited these changes; the paraprobiotic preserved colon length, reversed splenomegaly and regulated interleukin (IL)‐6/IL‐10 more effectively than the live probiotic (*P* < 0.05). All elevated PANoptosis markers (NLRP3, c‐GSDMD, c‐IL‐1*β*, p‐RIPK3, p‐MLKL, c‐caspase‐3, c‐GSDME and ZBP1) were suppressed by both treatments. ZBP1 was suppressed significantly more by the paraprobiotic (*P* < 0.05), which also increased butyric and valeric acid.

**CONCLUSION:**

The *L. acidophilus* LA‐5 paraprobiotic exerts protective effects at least equivalent to the live probiotic, supporting its potential as a safe therapeutic candidate for UC. © 2026 The Author(s). *Journal of the Science of Food and Agriculture* published by John Wiley & Sons Ltd on behalf of Society of Chemical Industry.

## INTRODUCTION

Ulcerative colitis (UC) is an inflammatory bowel disease (IBD) characterised by chronic, relapsing inflammation of the colonic and rectal mucosa with alternating periods of remission and flare. With symptoms such as abdominal pain, bloody diarrhoea and faecal urgency, UC markedly reduces quality of life and, over the long term, increases the risk of colorectal cancer.^1^ Over the past decade IBD has become a global public‐health problem. Although its incidence has plateaued in Western countries that traditionally show high prevalence, it is rising rapidly in industrialising nations.^1^, ^2^ Large‐scale health‐record studies comparing Japan and the USA have shown that UC prevalence increased significantly in both countries over a 10‐year period.^3^ Likewise, systematic analyses of worldwide trends in hospitalisations for Crohn's disease and UC in the twenty‐first century indicate that the disease burden continues to grow.^4^ These trends underscore the need for new therapeutic strategies when current treatments prove insufficient.

Although the pathogenesis of UC has not been fully elucidated, it is accepted to arise from a complex interplay of genetic predisposition, environmental factors, gut microbiota dysbiosis and dysregulation of the mucosal immune response.^5^ Reactive oxygen species (ROS) overproduced in the colonic epithelium lie at the centre of this process. Although ROS participate in cellular signalling under physiological conditions, in an inflammatory milieu, they become a pathological burden once they exceed the capacity of the enzymatic [superoxide dismutase (SOD); catalase (CAT)] and non‐enzymatic [glutathione (GSH)] antioxidant defences; by causing oxidative damage to lipids, proteins and nucleic acids, they disrupt epithelial integrity, trigger the release of pro‐inflammatory cytokines and aggravate inflammation in a vicious cycle.^6^ Indeed, deficiency of the antioxidant defence system (decreased SOD, CAT and GSH) together with increases in the lipid‐peroxidation product malondialdehyde (MDA) and the neutrophil‐infiltration marker myeloperoxidase (MPO) constitutes the characteristic biochemical signature of UC.^6^, ^7^ More importantly, mitochondria‐derived ROS mediate not only macromolecular damage, but also the activation of programmed cell‐death pathways, directly linking oxidative stress to epithelial cell death and barrier dysfunction and making this step a critical junction in UC pathophysiology.^7^, ^12^

In recent years, it has become clear that epithelial cell death in UC cannot be explained by classical apoptosis alone; pyroptosis, apoptosis and necroptosis are engaged simultaneously. Defined in 2019, PANoptosis is a specialised inflammatory cell‐death modality that combines the molecular features of these three programmed cell‐death pathways.^8^ PANoptosis is executed through the assembly of a multiprotein complex termed the ‘PANoptosome’, in which sensors such as AIM2, pyrin and Z‐DNA‐binding protein 1 (ZBP1) come together with effectors such as ASC, caspase‐1, caspase‐8, RIPK1, RIPK3 and FADD.^9^ ZBP1, which senses nucleic acids and mitochondrial ROS signals through its Z*α* domain to initiate PANoptosome assembly, is a key sensor and plays a central role in regulating cytokine storm and inflammatory cell death.^10^, ^11^ Mechanistically, caspase activation drives apoptosis (caspase‐3, GSDME), cleavage of gasdermin D (GSDMD) and activation of the NLRP3 inflammasome drive pyroptosis [interleukin (IL)‐1*β* release], and phosphorylation of the RIPK3‐MLKL axis drives necroptosis.^8^, ^9^ The convergence of these three pathways on a common molecular platform explains why interventions targeting a single cell‐death type are often insufficient and why suppression of the PANoptosome, particularly at the level of upstream sensors such as ZBP1, may provide more comprehensive protection.^9^, ^10^

The role of PANoptosis in UC pathogenesis is being elucidated rapidly. Ye *et al*.^12^ reported that, in the epithelial cells of UC patients and experimental colitis models, Drp1‐mediated mitochondrial fission triggers ZBP1‐dependent PANoptosis through ROS production and that this process correlates positively with disease severity. Bioinformatic and experimental validation studies have shown that key PANoptosis‐related genes in UC (ZBP1, AIM2, CASP1/8, IRF1) are associated with immune‐cell infiltration and disease severity.^13^, ^14^, ^15^, ^16^ Machine‐learning‐based analyses have likewise proposed that PANoptosis markers, ZBP1 in particular, may have diagnostic and classificatory value in UC.^16^ Moreover, the demonstration that natural compounds such as diosmin alleviate colitis by suppressing epithelial PANoptosis supports the therapeutic targetability of this pathway.^17^ The NLRP3‐GSDMD axis, the executive arm of pyroptosis, has also been independently confirmed to be hyperactivated in the UC mucosa and to drive macrophage and epithelial pyroptosis.^18^, ^19^, ^20^, ^21^ Suppression of PANoptosome components therefore offers a promising target for reducing epithelial cell death and achieving remission in UC.^22^, ^23^

Current UC treatment relies on aminosalicylates, glucocorticoids, immunosuppressants and biological agents; however, a substantial proportion of these therapies is limited by side effects (osteoporosis, nephrotoxicity, opportunistic infections) and by primary/secondary non‐response.^5^ Complementary strategies targeting the gut microbiota and mucosal homeostasis have therefore gained importance. Probiotics have been found effective in alleviating UC by strengthening the intestinal barrier, regulating microbial balance and modulating the immune response.^5^, ^33^, ^34^, ^35^, ^36^ *Lactobacillus* species in particular have been shown to exert anti‐inflammatory effects *via* Toll‐like receptor (TLR) signalling and macrophage polarisation, to increase tight‐junction proteins (ZO‐1, occludin) and to support short‐chain fatty acid (SCFA) production.^34^, ^35^ Support of SCFA production reinforces this protective effect by enhancing colonocyte energy metabolism and regulatory T‐cell responses.^36^, ^40^ Nevertheless, the capacity of live probiotics to cause bacteraemia, fungaemia and sepsis in immunocompromised individuals, premature neonates and patients with a weakened mucosal barrier is an important safety concern.^24^

These limitations have brought non‐viable microbial preparations to the fore. In 2021, the International Scientific Association of Probiotics and Prebiotics (ISAPP) defined the term ‘postbiotic’ as a ‘preparation of inanimate microorganisms and/or their components that confers a health benefit on the host’.^24^ Within this framework, paraprobiotics (‘inactivated’ or ‘ghost’ probiotics) denote non‐viable intact or disrupted microbial cells and are most often obtained by heat inactivation.^25^, ^26^ Paraprobiotics stand out as a safe alternative because they eliminate the risk of bacterial translocation and horizontal transfer of antibiotic‐resistance genes, offer advantages in shelf life and standardisation, and can exert immunomodulatory effects similar to those of live cells.^25^, ^26^, ^27^ These properties provide a critical safety advantage, particularly in immunocompromised patient groups.^24^, ^27^ Heat‐inactivated lactic acid bacteria have been reported to retain their antioxidant and immunostimulatory capacities,^28^ to display genoprotective and anti‐inflammatory effects,^29^ and to alleviate disease in experimental colitis models by regulating autophagy and barrier pathways.^30^, ^31^, ^32^

Despite this evidence, important gaps remain. First, the effects on AA‐induced UC of a paraprobiotic derived from *Lactobacillus acidophilus* LA‐5, a strain widely used in the food and supplement sector, have not been investigated. Second, and more importantly, to the best of our knowledge, no study has evaluated the effect of a paraprobiotic on the PANoptosis cell‐death pathway. Third, the effect of paraprobiotics on the caecal SCFA profile in UC is unknown. Considered together, these three gaps reveal a clear need to be filled, both for a safe therapeutic candidate and for a novel cell‐death target. Accordingly, this study aimed to evaluate *in vivo* the antioxidant, anti‐inflammatory and PANoptosis‐regulating effects of a heat‐inactivated paraprobiotic derived from *L. acidophilus* LA‐5 in AA‐induced UC, and to compare these effects with those of the live probiotic. We hypothesised that the paraprobiotic would alleviate colitis symptoms at least as effectively as the live probiotic by suppressing oxidative stress and ZBP1‐mediated PANoptosis, and that, as a result of its safety profile, it would constitute a more advantageous therapeutic candidate.

## MATERIALS AND METHODS

### Paraprobiotic production and verification of inactivation

The probiotic strain *L. acidophilus* LA‐5 (Chr. Hansen, Copenhagen, Denmark) was used to obtain the paraprobiotic. The strain was propagated in MRS broth at 37 °C for 24 h under anaerobic conditions to a viability of 10^8^ colony‐forming units (CFU) mL^−1^. Cells were pelleted by centrifugation (7000 × *g* at 4 °C for 10 min), washed three times with phosphate‐buffered saline (PBS) and heat‐treated in sterile PBS at 65 °C for 30 min in a water bath.

Loss of cell viability was confirmed by BD LSRFortessa™ flow cytometry (BD Biosciences, San Jose, CA, USA). A LIVE/DEAD BacLight kit (Thermo Fisher Scientific, Waltham, MA, USA) containing SYTO9 (staining live cells) and propidium iodide (PI; staining dead/membrane‐compromised cells) was used. After staining, samples were read on the SSC‐H/FL1‐H channels; gating was performed relative to a live control tube and analyses were carried out with FlowJo software (Fig. 1). Absence of colony growth was additionally confirmed by conventional plating on MRS agar. The inactivation and verification approach was consistent with a method previously described for the same strain.^41^

**Figure 1:**
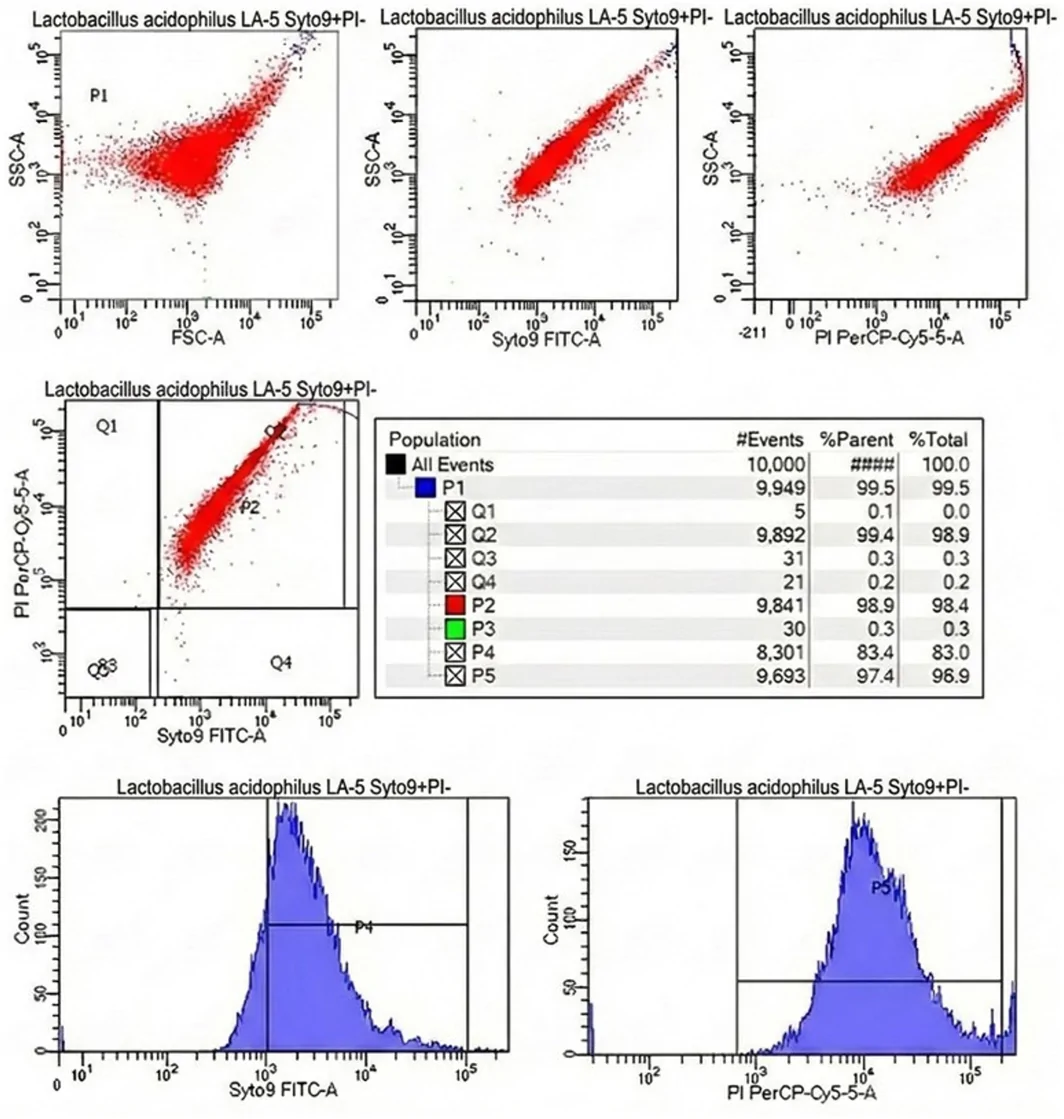
Verification of the heat inactivation of the *L. acidophilus* LA‐5 paraprobiotic by flow cytometry. SYTO9 (live cells, FITC channel) and propidium iodide (PI; dead/membrane‐compromised cells, PerCP‐Cy5.5 channel) double staining. Top row: cell gating with FSC‐A/SSC‐A and SYTO9/PI scatter plots; middle row: quadrant analysis and population statistics; bottom row: SYTO9 and PI fluorescence‐intensity histograms. More than 95% of cells were identified as PI‐positive (non‐viable).

### Animals, groups and colitis model

Ethical approval for the study was obtained from the relevant Aydın Adnan Menderes University Animal Experiments Local Ethics Committee (Protocol No. 64583101/2025/063). Forty female Balb/c mice aged 6–8 weeks were housed at 22 ± 1 °C under a 12:12 h light/dark photocycle with free access to standard pellet feed and water. Animals were randomly assigned to four groups with similar baseline weights (*n* = 10 per group) (Table 1).

**Table 1:** Experimental groups, colitis‐induction protocol and treatment regimens

Group (*n* = 10)	Abbreviation	Colitis induction (intrarectal)	Treatment regimen (oral gavage, 0.5 mL)
Healthy control	Control	Isotonic saline	Isotonic saline (0.5 mL)
Acetic acid	AA	4% acetic acid (100 μL)	Isotonic saline (0.5 mL)
Live probiotic	AA‐PRO	4% acetic acid (100 μL)	Live L. acidophilus LA‐5 (1 × 10^8^ CFU mL^−1^)
Paraprobiotic	AA‐PARA	4% acetic acid (100 μL)	Heat‐inactivated L. acidophilus LA‐5 (1 × 10^8^ CFU mL^−1^)

Colitis was induced under ketamine/xylazine anaesthesia by intrarectal administration of 4% acetic acid through a soft paediatric catheter; after administration the animals were held head‐down for 30 s to prevent reflux. Treatments were administered by oral gavage for 7 days starting the day after colitis induction.^42^

### Clinical evaluation

Animals were scored daily for body‐weight loss (%), stool consistency and faecal blood, and the disease activity index (DAI) was calculated as the mean of these three parameters.^42^ At the end of the experiment, cardiac blood samples, colon tissue and caecal contents were collected, and colon length and spleen weight were measured. Tissue samples were placed in 10% formaldehyde for histopathology and stored at −80 °C for biochemical and molecular analyses.

### Histopathological analysis

Colon tissues were embedded in paraffin after routine processing, and 5 μm sections were stained with haematoxylin‐eosin (H&E). Inflammation and tissue damage were evaluated by a blinded observer using a semi‐quantitative scoring system that took into account mucosal infiltration, epithelial damage and architectural distortion.^43^

### Enzyme‐linked immunosorbent assay (ELISA) of serum inflammatory markers

In serum obtained from cardiac blood, the systemic inflammation markers tumour necrosis factor‐*α* (TNF‐*α*, E‐CL‐M0047), interleukin‐6 (IL‐6, E‐EL‐M0044), interleukin‐1*β* (IL‐1*β*, E‐EL‐M0037), the anti‐inflammatory cytokine interleukin‐10 (IL‐10, E‐EL‐M0046) and C‐reactive protein (CRP, E‐EL‐M0053) were measured with commercial ELISA kits in accordance with the manufacturer's instructions (Elabscience Bionovation Inc., Houston, TX, USA) on a microplate reader (Thermo Fisher Scientific). Results were expressed as pg mL^−1^ for TNF‐*α*, IL‐6 and IL‐1*β*, and as ng mL^−1^ for IL‐10 and CRP.

### Oxidant/antioxidant biomarkers

In colon‐tissue homogenates, MDA was measured by the thiobarbituric acid reactive substances (i.e. TBARS) method (532 nm)^45^; CAT activity from the decreasing UV absorbance of hydrogen peroxide (240 nm); GSH by the 5,5′‐dithiobis‐(2‐nitrobenzoic acid) (i.e. DTNB) enzymatic recycling method (412 nm); and MPO activity spectrophotometrically with an *o*‐dianisidine/hydrogen peroxide substrate (450 nm).^44^ All measurements were expressed per unit protein.^43^

### Western blot analysis of PANoptosis proteins

Colon tissue (50 mg) was homogenised in ice‐cold RIPA buffer (Thermo Fisher Scientific) containing protease/phosphatase inhibitors; 40 μg of protein was separated by sodium dodecyl sulfate–polyacrylamide gel electrophoresis and transferred to polyvinylidene fluoride membranes (Millipore, Merck, Darmstadt, Germany). After blocking with 5% non‐fat milk, membranes were incubated overnight at 4 °C with primary antibodies (Cell Signaling Technology, Danvers, MA, USA; dilution 1:1000) against PANoptosis‐related targets (IL‐1*β*, c‐IL‐1*β*, GSDMD, c‐GSDMD, caspase‐3, RIP3, p‐RIP3, MLKL, p‐MLKL, GSDME, c‐GSDME, NLRP3, ZBP1), with *β*‐actin as loading control. After incubation with horseradish protein‐conjugated secondary antibodies (dilution 1:5000, 1 h at room temperature), bands were visualised with ECL substrate on a Syngene G:BOX Chemi XX9 imaging system (Syngene, Cambridge, UK) and quantified in ImageJ (MIH, Bethesda, MD, USA) normalised to *β*‐actin.^42^

### Determination of short‐chain fatty acids

SCFAs were extracted from caecal contents using a modified protocol based on Calderón‐Pérez *et al*.^46^ Briefly, 0.1 g of caecal content was homogenised in 1% HCl containing 500 μm 4‐methylvaleric acid as internal standard; the mixture was agitated for 15 min (1500 rpm) and subjected to two‐stage centrifugation at 4 °C (1800 × *g* for 10 min, then the supernatant at 8784 × *g* for 4 min), and the clear phase was passed through a 0.45‐μm cellulose acetate filter. SCFA profiles were determined by gas chromatography with flame‐ionisation detection (GC‐FID; model 7697A; Agilent, Santa Clara, CA, USA) fitted with a high‐polarity DB‐WAX capillary column (30 m × 0.25 mm × 0.25 μm; Agilent); nitrogen was the carrier gas, the injection volume was 1 μL (split), and the injector and detector temperatures were 260 °C and 280 °C, respectively. The oven programme started at 85 °C (1 min), was increased at 5 °C min^−1^ to 160 °C (1 min) and then at 40 °C min^−1^ to 240 °C (2 min). Quantification was performed with the WSFA‐2 reference standard mixture (WSFA‐2 Mixture; Sigma‐Aldrich) and the results are reported in parts per million (ppm).

### Statistical analysis

All analyses were performed using Prism, version 10.6.1 (GraphPad Software Inc., San Diego, CA, USA). The normality of data distribution was assessed; one‐way analysis of variance followed by Tukey's multiple‐comparison test was used for normally distributed data with homogeneous variances, and the Kruskal–Wallis test followed by Dunn's test for non‐normally distributed data. Data are presented as mean ± SD. *P* < 0.05 was considered statistically significant.

## RESULTS

### Heat inactivation confirmed by flow cytometry

After the 65 °C/30 min heat treatment of the *L. acidophilus* LA‐5 strain, flow‐cytometry analysis showed that the overwhelming majority of the cell population had lost membrane integrity and was stained with PI (Fig. 1). In SYTO9/PI double staining, the proportion of dead cells exceeded 95%, a result supported by the absence of colony growth on conventional MRS agar plating. The preparation used in the animal experiments was thus confirmed to be a genuine paraprobiotic (non‐viable cells).

### The paraprobiotic alleviated colitis symptoms and reversed splenomegaly

Following colitis induction, the DAI rose rapidly in the AA group, reaching its highest level at the end of the experiment, whereas no signs of disease were observed in the control group (Fig. 2(A)). In both the AA‐PRO and AA‐PARA groups, the DAI declined markedly from day 3 onward, with the lowest values recorded in the paraprobiotic group. Similarly, body weight fell to approximately 83% of baseline in the AA group, whereas the paraprobiotic and probiotic treatments significantly limited weight loss (96% and 93%, respectively) (Fig. 2(B)).

**Figure 2:**
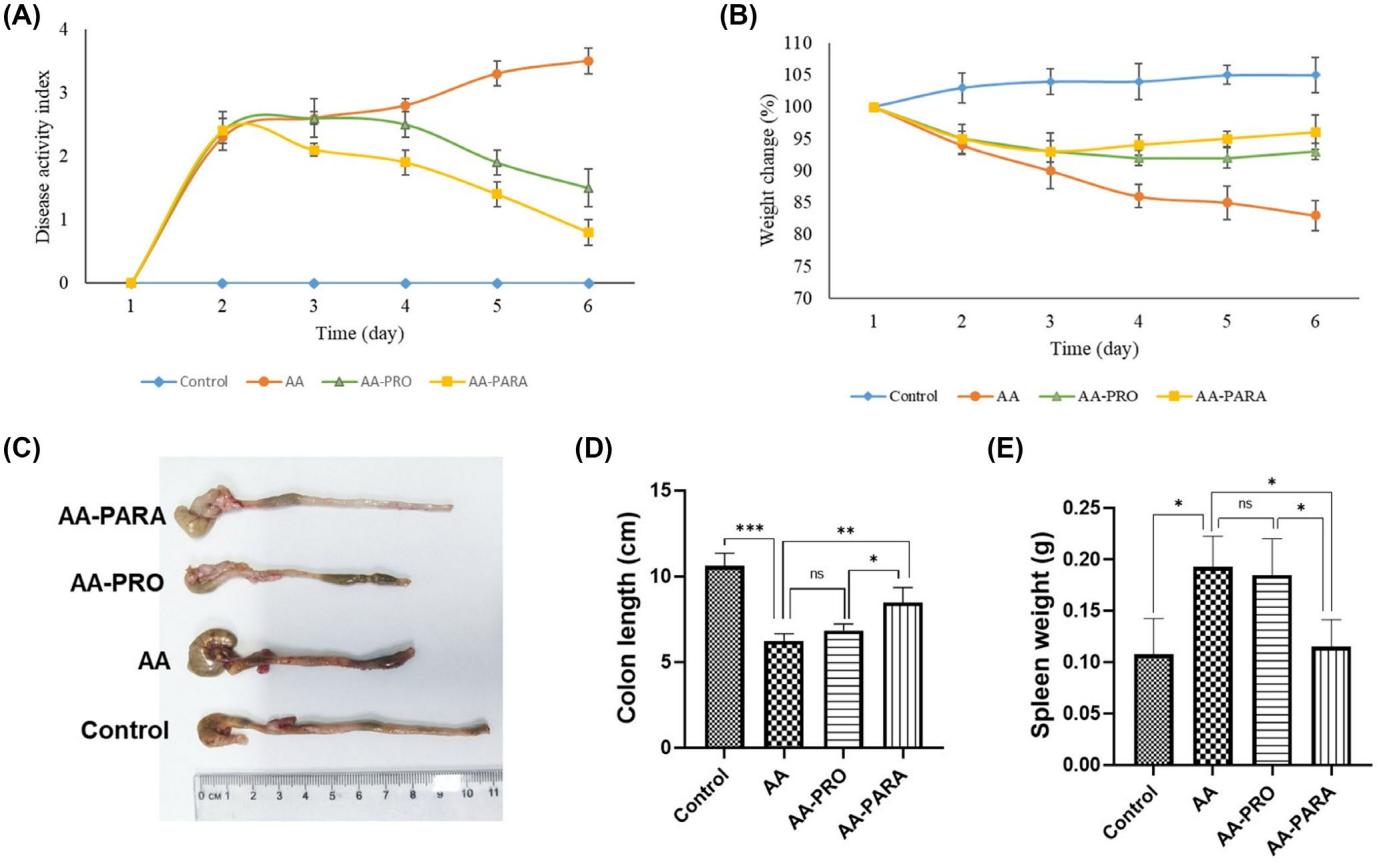
Effect of probiotic and paraprobiotic treatment on the clinical and macroscopic findings of colitis. (A) Daily disease activity index (DAI). (B) body‐weight change relative to baseline (%). (C) representative macroscopic colon images. (D) colon length (cm). (E) spleen weight (g). Data are the mean ± SD (*n* = 10). *P < 0.05, **P < 0.01, ***P < 0.001; ns, not significant.

Colon shortening, an important indicator of colitis severity, was pronounced in the AA group (6.2 cm *versus* 10.5 cm in controls; *P* < 0.001) (Fig. 2(C),(D)). Paraprobiotic treatment significantly preserved colon length (8.5 cm; *P* < 0.01 *versus* AA) and was statistically more effective than the live probiotic (6.8 cm) (*P* < 0.05 *versus* AA‐PRO). By contrast, the effect of the live probiotic on colon length did not differ significantly from the AA group (*P* > 0.05). Spleen weight, an indicator of systemic inflammation, was increased in the AA group (splenomegaly; *P* < 0.05 *versus* control); paraprobiotic treatment returned spleen weight to the control level (*P* < 0.05 *versus* AA) and this effect was again more pronounced than that of the live probiotic (*P* < 0.05 *versus* AA‐PRO), whereas the live probiotic did not significantly reduce splenomegaly (Fig. 2(E)).

### Both treatments reduced histopathological damage

In the control group, the colonic mucosa showed normal crypt architecture and intact epithelium, whereas the AA group showed extensive epithelial loss, crypt destruction, oedema and dense inflammatory‐cell infiltration (Fig. 3(A)). In both the AA‐PRO and AA‐PARA groups, the mucosal architecture was markedly preserved. The histopathological damage score was highest in the AA group (7.3); paraprobiotic (3.8) and probiotic (4.3) treatments significantly reduced this score (*P* < 0.01 *versus* AA for both), with no significant difference between the two treatments (*P* > 0.05, Fig. 3(B)). MPO activity, an indicator of neutrophil infiltration, increased approximately tenfold in the AA group (105 *versus* 10 nmol min^−1^ mg^−1^ protein in controls; *P* < 0.001); both treatments significantly suppressed MPO (AA‐PRO 53 and AA‐PARA 40 nmol min^−1^ mg^−1^ protein; *P* < 0.001 for both) (Fig. 3(C)).

**Figure 3:**
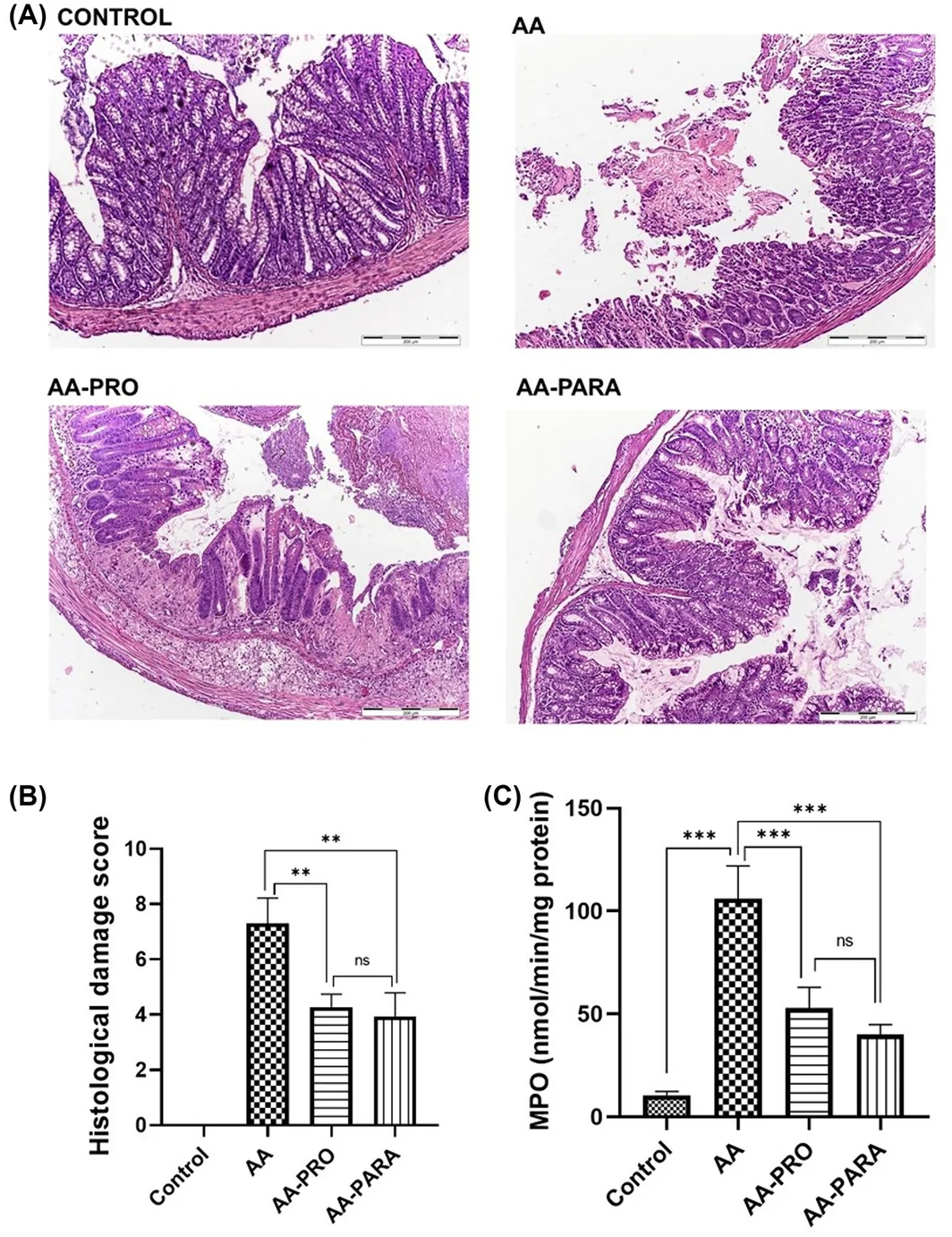
Histopathological evaluation and neutrophil infiltration. (A) Representative H&E‐stained colon sections from the Control, AA, AA‐PRO and AA‐PARA groups; the AA group shows extensive epithelial loss, crypt destruction and inflammatory infiltration, with marked improvement in the treatment groups. (B) Histopathological damage score. (C) myeloperoxidase (MPO) activity (nmol min^−1^ mg^−1^ protein). Data are the mean ± SD. **P < 0.01, ***P < 0.001; ns, not significant.

### The paraprobiotic restored antioxidant capacity and reduced oxidative damage

In the AA group, the enzymatic (SOD, CAT) and non‐enzymatic (GSH) antioxidant defences were markedly depleted (Fig. 4). SOD activity fell from 61 to 18 nmol mg^−1^ protein (*P* < 0.001), CAT fell from 49 to 18.5 nmol mg^−1^ protein (*P* < 0.001) and GSH fell from 1.74 to 0.76 nmol mg^−1^ protein (*P* < 0.001). In parallel, the lipid‐peroxidation product MDA increased approximately threefold in the AA group (from 11 to 30 nmol mg^−1^ protein; *P* < 0.001). Both probiotic and paraprobiotic treatments significantly increased SOD, CAT and GSH and lowered MDA (*P* < 0.05–0.01 *versus* AA). For all antioxidant parameters, the difference between the paraprobiotic and the probiotic was not statistically significant (*P* > 0.05), indicating that the paraprobiotic exerts antioxidant activity equivalent to that of the live probiotic.

**Figure 4:**
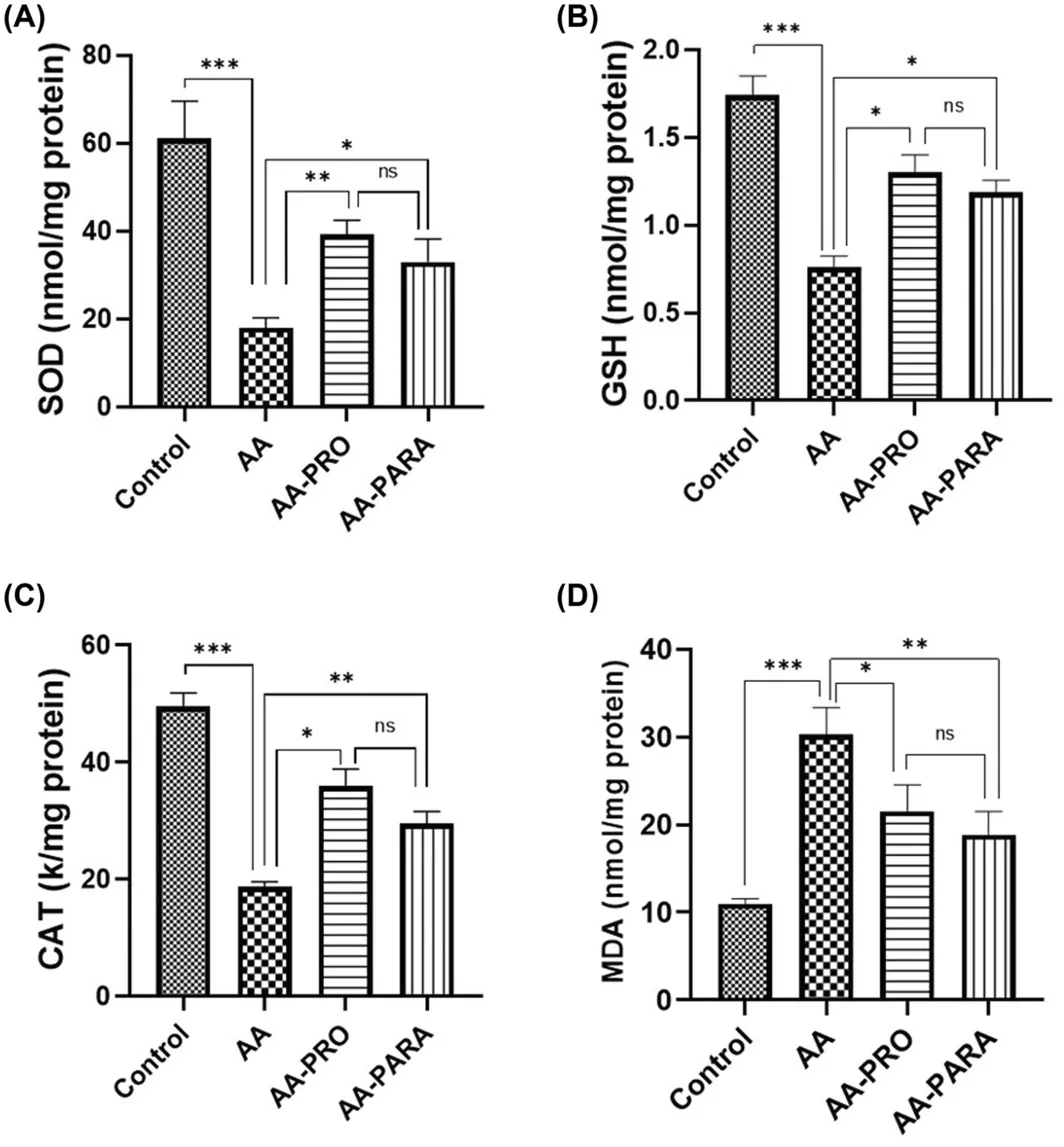
Oxidant/antioxidant biomarkers in colon tissue. (A) superoxide dismutase (SOD). (B) glutathione (GSH). (C) catalase (CAT). (D) malondialdehyde (MDA). All values expressed per unit protein. Data are the mean ± SD. *P < 0.05, **P < 0.01, ***P < 0.001; ns, not significant.

### The paraprobiotic reduced systemic pro‐inflammatory cytokines and increased IL ‐10

Serum ELISA analyses showed that the systemic inflammatory load was markedly increased in the AA group (Fig. 5). The pro‐inflammatory cytokines TNF‐*α* (from 55 to 505 pg mL^−1^; *P* < 0.001), IL‐6 (from 30 to 545 pg mL^−1^; *P* < 0.001) and IL‐1*β* (from 15 to 258 pg mL^−1^; *P* < 0.001), together with CRP (from 46 000 to 78 000 ng mL^−1^; *P* < 0.001), were elevated in the AA group, whereas the anti‐inflammatory cytokine IL‐10 was markedly decreased (from 60 to 19 ng mL^−1^; *P* < 0.001).

**Figure 5:**
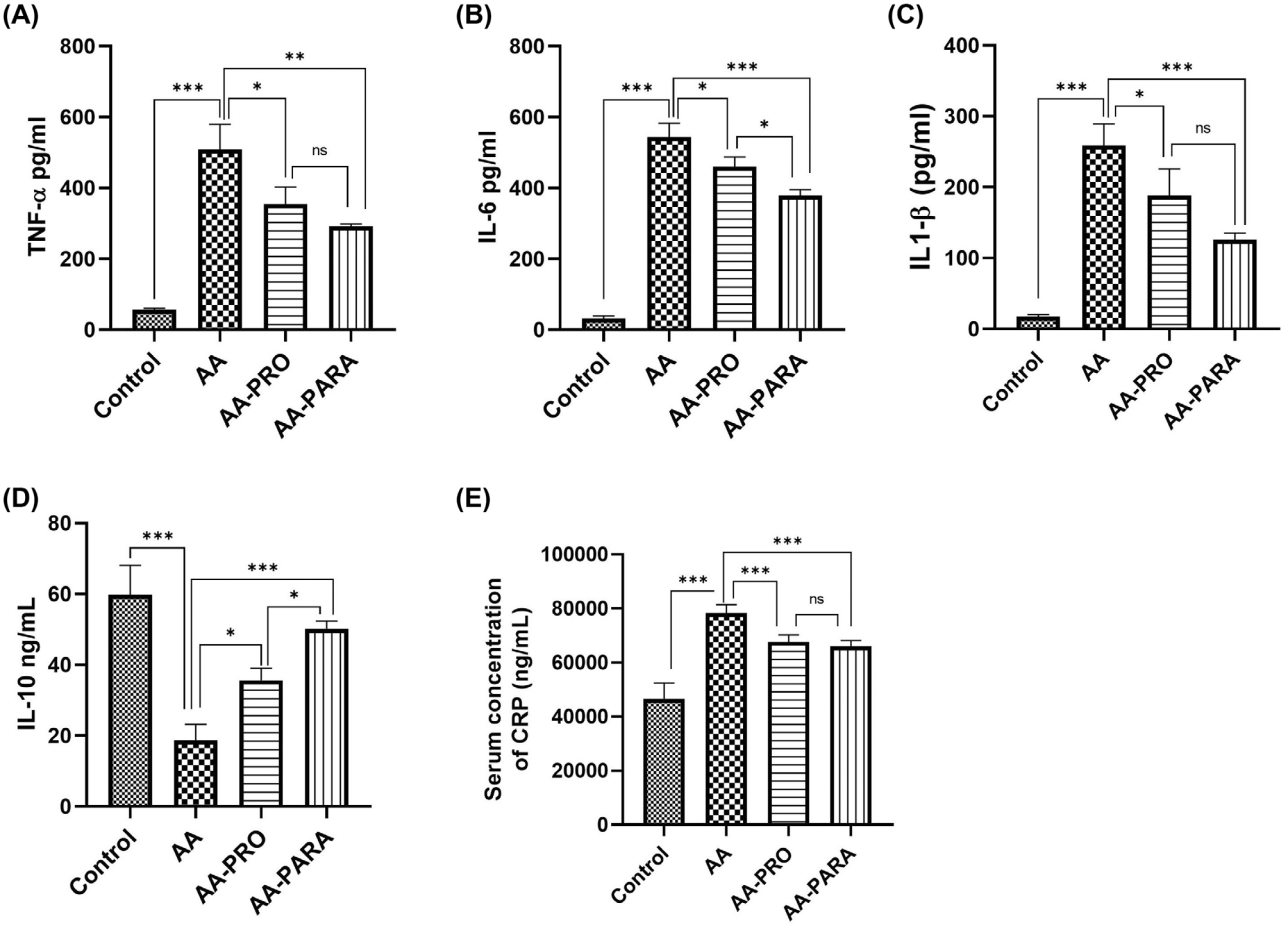
ELISA evaluation of serum inflammatory markers. (A) TNF‐*α* (pg mL^−1^). (B) IL‐6 (pg mL^−1^). (C) IL‐1*β* (pg mL^−1^). (D) anti‐inflammatory IL‐10 (ng mL^−1^). (E) C‐reactive protein, CRP (ng mL^−1^). Pro‐inflammatory cytokines and CRP increased and IL‐10 decreased in the AA group; both treatments reversed this profile, and the paraprobiotic was more effective than the live probiotic in regulating IL‐6 and IL‐10. Data are the mean ± SD. *P < 0.05, **P < 0.01, ***P < 0.001; ns, not significant.

Both probiotic and paraprobiotic treatments favourably corrected this inflammatory profile: they significantly reduced TNF‐*α*, IL‐6, IL‐1*β* and CRP and increased IL‐10 (*P* < 0.05–0.001 *versus* AA). The paraprobiotic was significantly more effective than the live probiotic in regulating IL‐6 (375 pg mL^−1^) and the anti‐inflammatory IL‐10 (50 ng mL^−1^) (*versus* 460 pg mL^−1^ and 35 ng mL^−1^, respectively; *P* < 0.05 *versus* AA‐PRO); IL‐1*β* also fell to its lowest level in the paraprobiotic group (127 pg mL^−1^). No significant difference between the two treatments was observed for TNF‐*α* or CRP (*P* > 0.05). These findings suggest that, in addition to its local antioxidant effect, the paraprobiotic can restore systemic inflammatory balance to an extent equivalent to, or for some markers greater than, the live probiotic.

### The paraprobiotic suppressed the PANoptosis pathway and inhibited ZBP1 more strongly than the probiotic

Western blot analyses revealed that pyroptosis, apoptosis and necroptosis markers were simultaneously and markedly increased in the AA group, indicating activation of PANoptosis (Fig. 6). Along the pyroptosis axis, NLRP3 (*P* < 0.001), the cleaved‐GSDMD/GSDMD ratio (*P* < 0.001) and the cleaved‐IL‐1*β*/IL‐1*β* ratio (*P* < 0.001) were strongly elevated in the AA group relative to controls. Along the necroptosis axis, the p‐RIPK3/RIPK3 (*P* < 0.001) and p‐MLKL/MLKL (*P* < 0.001) ratios, and along the apoptosis axis, the cleaved‐caspase‐3/caspase‐3 (*P* < 0.001) and cleaved‐GSDME/GSDME (*P* < 0.001) ratios, were markedly increased.

**Figure 6:**
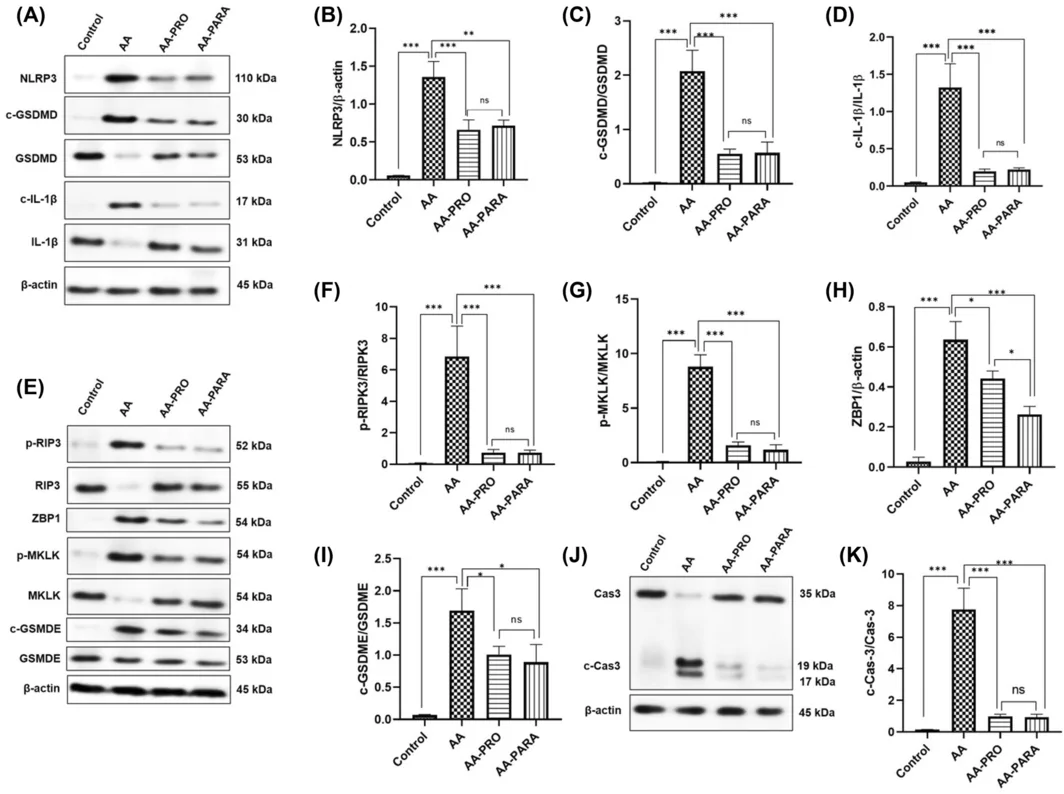
Western blot analysis of PANoptosis‐pathway proteins in colon tissue. (A) Representative blots of pyroptosis markers (NLRP3, c‐GSDMD, GSDMD, c‐IL‐1*β*, IL‐1*β*, *β*‐actin). (B) NLRP3/*β*‐actin. (C) c‐GSDMD/GSDMD. (D) c‐IL‐1*β*/IL‐1*β*. (E) Blots of necroptosis and additional markers (p‐RIPK3, RIPK3, ZBP1, p‐MLKL, MLKL, c‐GSDME, GSDME, *β*‐actin). (F) p‐RIPK3/RIPK3. (G) p‐MLKL/MLKL. (H) ZBP1/*β*‐actin, the paraprobiotic suppressed ZBP1 significantly more strongly than the live probiotic. (I) c‐GSDME/GSDME. (J) Apoptosis‐marker blot (caspase‐3, c‐caspase‐3, *β*‐actin). (K) c‐caspase‐3/caspase‐3. Band intensities were normalised to *β*‐actin. Data are the mean ± SD. *P < 0.05, ***P < 0.001; ns, not significant.

Both probiotic and paraprobiotic treatments significantly suppressed all of these markers (*P* < 0.05–0.001 *versus* AA). Although no significant difference between the two treatments was found for most PANoptosis markers (*P* > 0.05), the critical difference emerged for ZBP1: ZBP1 was markedly increased in the AA group (*P* < 0.001) and decreased with both treatments, but was suppressed significantly more strongly in the paraprobiotic group than with the live probiotic (AA‐PRO *versus* AA‐PARA; *P* < 0.05) (Fig. 6(H)). This finding suggests that the paraprobiotic may suppress the pathway from a relatively proximal point by targeting ZBP1, one of the upstream sensors of PANoptosis, and may in this respect exert a more pronounced effect than the live probiotic.

### The paraprobiotic increased caecal SCFA production

GC analysis of caecal contents showed that the major SCFAs were markedly decreased in the colitis group (Fig. 7). Acetic acid (from 555 to 380 ppm; *P* < 0.001), butyric acid (from 255 to 155 ppm; *P* < 0.001), propionic acid (from 74 to 42 ppm; *P* < 0.01) and valeric acid (from 47 to 25 ppm; *P* < 0.001) decreased in the AA group, whereas the branched‐chain isobutyric and isovaleric acid levels did not differ between groups (*P* > 0.05). Both probiotic and paraprobiotic treatments significantly increased acetic, propionic, butyric and valeric acid. Strikingly, the paraprobiotic increased butyric (322 ppm) and valeric acid (41 ppm) production significantly higher than the live probiotic (*P* < 0.001 and *P* < 0.05 *versus* AA‐PRO); the butyric acid level in the paraprobiotic group even exceeded that of the healthy control. This result suggests that non‐viable cells too can favourably modulate gut microbial metabolism.

**Figure 7:**
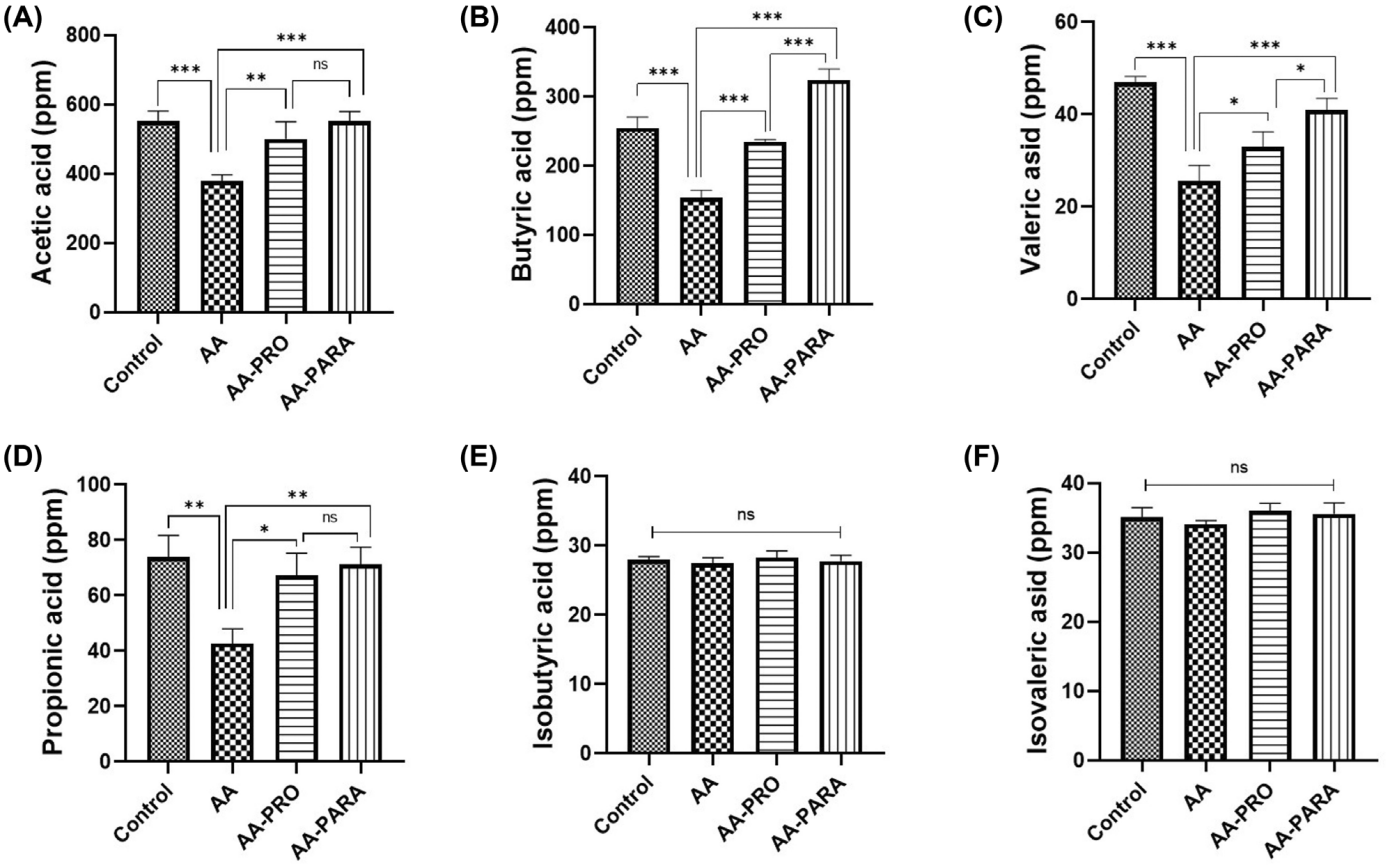
Caecal short‐chain fatty acid (SCFA) profile (GC analysis, ppm). (A) acetic acid. (B) butyric acid. (C) valeric acid. (D) propionic acid. (E) isobutyric acid. (F) isovaleric acid. The paraprobiotic increased butyric and valeric acid production higher than the live probiotic. Data are the mean ± SD. *P < 0.05, **P < 0.01, ***P < 0.001; ns, not significant.

## DISCUSSION

To the best of our knowledge, this study is among the first *in vivo* studies to evaluate the effects of a heat‐inactivated paraprobiotic derived from *L. acidophilus* LA‐5 on acetic acid‐induced ulcerative colitis and the possible effect of a paraprobiotic on the PANoptosis cell‐death pathway. The findings show that the paraprobiotic exhibits a consistent protective profile at clinical, histopathological, biochemical, immunological, molecular and metabolomic levels, and that this profile largely overlaps with that of the live probiotic. The systematic investigation of paraprobiotic‐based approaches in UC is relatively recent; our findings therefore contribute to the field by offering a mechanistic perspective that is both strain‐specific and directed at a cell‐death pathway. These findings are discussed below in the context of their possible mechanisms and their relationship with the literature.

The consistent induction of weight loss, colon shortening, splenomegaly, dense neutrophil infiltration (increased MPO) and mucosal damage by the acetic acid model confirms its validity in mimicking human UC. The reduction of DAI and limitation of colon shortening and weight loss by the probiotic and paraprobiotic interventions are consistent with numerous probiotic colitis studies.^33^, ^34^, ^35^, ^36^, ^38^ *Lactobacillus* species are widely documented to act by preserving barrier integrity, restoring microbial balance and regulating the immune response in intestinal diseases.^33^ Wu *et al*.^35^ showed that *Lactiplantibacillus plantarum* HNU082 preserved body weight, colon length and the mucosal barrier and reduced MPO and pro‐inflammatory cytokines in dextran sulfate sodium (DSS) colitis, whereas Li *et al*.^38^ reported that different *Lactobacillus* strains acting cooperatively alleviated DSS colitis. In our results, the more pronounced effect of the paraprobiotic in preserving colon length and reversing splenomegaly compared to the live probiotic is noteworthy because spleen enlargement is an indicator of systemic inflammatory load and suggests that the paraprobiotic can limit not only the local, but also the systemic inflammatory response. This difference may be related to cell‐wall components (peptidoglycan, lipoteichoic acid) and cell‐surface proteins (p40/p75‐like molecules) released during heat inactivation entering into a more standardised and predictable interaction with the host immune system.^32^ Indeed, Sun *et al*.^30^ reported that live and heat‐killed *Limosilactobacillus reuteri* cells regulated the colonic immune response differently in experimental colitis but both in a protective direction, and Rezaie *et al*.^31^ showed that a novel paraprobiotic cocktail controlled DSS colitis by regulating autophagy signalling.

In this context, accumulating evidence on the efficacy of paraprobiotics in experimental colitis places our findings in a broader framework. In a previous study by our group, a paraprobiotic derived from *Lacticaseibacillus casei* 39 was shown to favourably regulate inflammatory and oxidative pathways in the same acetic acid model^42^; the present study extends this observation to a different and industrially widely used strain (*L. acidophilus* LA‐5) and, for the first time, to the PANoptosis cell‐death pathway. Likewise, studies demonstrating that heat‐killed lactobacilli and paraprobiotic cocktails are protective in experimental colitis^29^, ^30^, ^31^ support the therapeutic potential of non‐viable cells. This consistency suggests that paraprobiotic efficacy is not a coincidence specific to a single strain but may be a common feature shared by non‐viable lactic acid bacteria.

Oxidative stress lies at the centre of UC pathogenesis; overproduction of ROS is a common mechanism linking epithelial damage, barrier dysfunction and inflammation.^6^, ^7^ In our study, the depletion of SOD, CAT and GSH and the increase in MDA in the AA group are in full agreement with this literature.^6^, ^39^ The restoration of antioxidant enzymes and reduction of lipid peroxidation by both probiotic and paraprobiotic treatments indicate that these preparations exert a protective effect by reducing the ROS load; similarly, Chen *et al*.^39^ reported that *Lactobacillus* protected the barrier by regulating the nuclear factor‐kappa B pathway and improving oxidative stress in colitis aggravated by a high‐salt diet. Importantly, the antioxidant activity of the paraprobiotic was indistinguishable from that of the live probiotic, consistent with studies reporting that heat treatment preserves antioxidant capacity. Hyun *et al*.^28^ showed that heat‐treated paraprobiotics retained antioxidant and immunostimulatory effects, and Kostelac *et al*.^29^ reported that probiotic and paraprobiotic derivatives exerted genoprotective and anti‐inflammatory effects against oxidative stress. Antioxidant protection may also indirectly limit cell death by reducing mitochondrial ROS, an upstream trigger of PANoptosis.^12^

The systemic inflammatory response that develops in parallel with oxidative stress is also a key determinant of UC severity. In our study, the increase in TNF‐*α*, IL‐6, IL‐1*β* and CRP and the suppression of anti‐inflammatory IL‐10 in the AA group confirm that the disease has a systemic inflammatory component. The restoration of this cytokine balance by both treatments is consistent with studies reporting that *Lactobacillus* species exert anti‐inflammatory effects *via* macrophage polarisation and TLR signalling^34^, ^35^; Jia *et al*.^34^ showed that *Lactobacillus johnsonii* increased IL‐10 release and alleviated colitis by activating CD206+ macrophages through the TLR1/2–STAT3 axis. The more pronounced effect of the paraprobiotic, particularly in lowering IL‐6 and increasing IL‐10 compared with the live probiotic, suggests that non‐viable cells too can possess considerable immunomodulatory capacity and that this effect may not depend solely on live‐cell metabolism. Given that IL‐6 is one of the principal drivers of the acute‐phase response and of CRP production, the more effective suppression of this cytokine also mechanistically supports the observed reduction in systemic inflammatory load. This systemic anti‐inflammatory shift also accounts for the observed reversal of splenomegaly. Given that TNF‐*α* is one of the main pro‐inflammatory signals triggering PANoptosome assembly,^8^ the reduction of the pro‐inflammatory cytokine load is also directly related to the suppression of PANoptosis discussed below.

One of the original contributions of our study is that it is among the first to address the possible regulatory effect of a paraprobiotic on the PANoptosis pathway. The simultaneous increase in pyroptosis (NLRP3, c‐GSDMD, c‐IL‐1*β*), necroptosis (p‐RIPK3, p‐MLKL) and apoptosis (c‐caspase‐3, c‐GSDME) markers in the AA group is consistent with current evidence showing activation of PANoptosis in UC.^12^, ^13^, ^14^, ^15^, ^16^, ^17^ Activation of the NLRP3 inflammasome and exposure of the pore‐forming N‐terminal fragment of GSDMD constitute a well‐defined axis that enables maturation and release of IL‐1*β* and drives epithelial and macrophage pyroptosis; blockade of this axis at the molecular or pharmacological level has been found protective in experimental colitis.^18^, ^19^, ^20^, ^21^ ZBP1, in turn, is well established as the key sensor initiating PANoptosome assembly.^9^, ^10^ The suppression of all three axes by both interventions suggests that *L. acidophilus* LA‐5 (whether live or non‐viable) can limit inflammatory cell death in a multifaceted manner. Notably, however, one of the striking findings is that the paraprobiotic suppressed ZBP1 significantly more strongly than the live probiotic. Because ZBP1 is one of the proximal regulators of PANoptosis,^10^, ^22^, ^23^ the more pronounced suppression of this sensor may contribute to a more comprehensive limitation of the downstream cell‐death axes. The demonstration by Ye *et al*.^12^ that ZBP1‐dependent PANoptosis correlates with disease severity in UC and that inhibition of this pathway can provide therapeutic benefit suggests that the more pronounced suppression of ZBP1 by the paraprobiotic may confer a clinical advantage. This relatively selective advantage on ZBP1 is thought to be related either to non‐viable cells triggering the endogenous nucleic acid/ROS signals that stimulate the Z*α* domain less than live cells do, or to a direct immunomodulatory effect of cell‐wall components; however, confirmation of this mechanism requires further molecular studies.

Another original aspect of our study is that it reveals the effect of the paraprobiotic on the caecal SCFA profile. SCFAs, butyrate in particular, are the principal energy source of colonocytes and have anti‐inflammatory, barrier‐strengthening and regulatory T‐cell‐inducing effects.^36^, ^40^ Given that butyrate induces regulatory T cells through histone deacetylase inhibition and supports colonocyte oxidative metabolism, the increase in this SCFA corresponds directly to an anti‐inflammatory gain.^40^ The reduction of SCFA production in UC is well documented, and butyrate supplementation has been shown to reduce inflammation in active UC in randomised controlled trials.^40^ In our results, the fact that the paraprobiotic increased butyric and valeric acid production even higher than the live probiotic is an unexpected and noteworthy finding. Because non‐viable cells cannot proliferate, this effect may be explained by paraprobiotic cell components modulating the host microbiota in a prebiotic‐like manner, particularly by supporting butyrate‐producing commensal bacteria. This is conceptually consistent with studies showing that polysaccharide‐ and probiotic‐based interventions increase SCFA production and beneficial taxa in DSS colitis.^36^, ^38^ The increase in SCFAs may be an important mechanism underlying the observed anti‐inflammatory and barrier‐protective effects and may form an integrated protective network together with the suppression of oxidative stress and PANoptosis.

Taken together, these findings indicate that the effect of the paraprobiotic cannot be reduced to a single mechanism; reduction of oxidative stress, restoration of systemic cytokine balance, increased SCFA production and suppression of ZBP1‐centred PANoptosis appear to form an interacting, integrated protective network. The fact that mitochondrial ROS mediate both direct oxidative damage and the activation of ZBP1 mechanistically justifies the link between the recovery of antioxidant capacity and the suppression of PANoptosis.^7^, ^12^ Similarly, SCFA‐mediated barrier strengthening and a regulatory immune response may indirectly limit inflammatory cell death by reducing epithelial exposure to death stimuli.^36^, ^40^ The paraprobiotic therefore appears to intervene simultaneously at different, mutually reinforcing nodes of UC pathophysiology.

These findings may be important from a clinical and translational standpoint. The bacteraemia/sepsis risk that live probiotics carry in immunocompromised patients^24^ is particularly relevant in a disease such as UC in which the mucosal barrier is already compromised. The fact that the paraprobiotic exerts effects equivalent to, or for some parameters greater than, the live probiotic and, because it contains no live cells, may reduce the risk of translocation and antibiotic‐resistance‐gene transfer^25^, ^26^, ^27^ highlights it as a safe therapeutic candidate with shelf‐life advantages. The clarification of the postbiotic definition by ISAPP^24^ reduces the obstacles to standardisation and regulatory approval of such preparations; that it is obtained from a strain such as LA‐5, which is widely used in the food and supplement sector and has a well‐defined safety record, provides an additional advantage for clinical translation. In this respect, paraprobiotics may offer a particularly valuable option in patient groups that do not respond to conventional treatments or in whom the use of live probiotics is risky because of immunosuppression.

From a translational perspective, the fact that paraprobiotics are standardisable, resistant to heat and shelf conditions, and have a more predictable safety profile because they carry no live cells may facilitate clinical development. The evaluation of *Lactobacillus*‐based interventions in an increasing number of clinical trials in digestive‐system diseases^37^ provides a suitable basis for testing such preparations in human UC. Nevertheless, determination of the effective dose, standardisation of the effect of the inactivation method (heat, ultrasonic or other) on biological activity, and demonstration of long‐term safety are key questions to be answered before clinical translation. Furthermore, elucidation of the dose‐ and time‐dependence of PANoptosis suppression, particularly the effect on ZBP1, will allow a more precise definition of the mechanism of action of the paraprobiotic.

Our study has some limitations. First, the acetic acid model is an acute colitis model and does not fully reflect the chronic, immune‐mediated nature of human UC; the findings need to be confirmed with DSS or chronic models and ultimately with clinical studies. Second, the molecular mechanism underlying the more pronounced suppression of ZBP1 (Z*α*‐domain binding, mitochondrial ROS, specific cell‐wall components) was not directly examined. Third, 16S rRNA or metagenomic analysis would be useful to determine which taxa are associated with the increase in SCFAs. In addition, the fact that the study was conducted only in female mice limits the generalisability of the results to both sexes and prevents the assessment of possible sex‐dependent differences. Despite these limitations, the consistent convergence of multiple independent endpoints (clinical, histological, biochemical, molecular, metabolomic) in the same direction supports the internal consistency of the results. In future studies, the comparison of different inactivation methods and dose ranges will help to identify the conditions that maximise paraprobiotic efficacy.

## CONCLUSIONS

The paraprobiotic obtained by heat inactivation of *L. acidophilus* LA‐5 can suppress colitis severity, oxidative stress and ZBP1‐mediated PANoptosis in acetic acid‐induced experimental ulcerative colitis. The paraprobiotic is largely equivalent to the live probiotic in antioxidant and anti‐inflammatory efficacy, and may exert a more pronounced effect in preserving colon length, reversing splenomegaly, suppressing ZBP1 and increasing butyric/valeric acid production. The consistent protective profile across multiple independent endpoints strengthens the reliability of these results. As a result of its lack of live cells, which reduces the risk of bacterial translocation and antibiotic‐resistance transfer and offers advantages in shelf life and standardisation, this paraprobiotic can be regarded as a safe and promising therapeutic candidate for UC. Nevertheless, elucidation of the molecular mechanism underlying the selective effect on ZBP1 and confirmation of the findings with chronic colitis models, microbiota analyses and ultimately clinical studies will make an important contribution to the development of paraprobiotic‐based therapies. Overall, with its potential to combine safety and efficacy, the heat‐inactivated *L. acidophilus* LA‐5 paraprobiotic warrants further investigation as a complementary treatment strategy for UC.

## FUNDING INFORMATION

The present research was financially sustained by the Scientific and Technological Research Council of Türkiye (TÜBİTAK) under the project grant framework No. 125Z143.

## CONFLICTS OF INTEREST

The authors declare that they have no conflicts of interest.

## ETHICS STATEMENT

All experimental protocols were executed in strict adherence to the ‘Guide for the Care and Use of Laboratory Animals’ and were formally approved by the Aydın Adnan Menderes University Animal Experiments Local Ethics Committee (Protocol No: 64583101/2025/063).

## AUTHOR CONTRIBUTIONS

AY was responsible for conceptualisation

AY and EA were responsible for methodology. AY and EÖ were responsible for investigations. AY was responsible for formal analysis and writing the original draft. EÖ was responsible for histopathological analysis. EÖ and EA were responsible for reviewing and editing. EA was responsible for resources and supervision. All authors read and approved the final version of the manuscript submitted for publication.

## Data Availability

The data that support the findings of this study are available from the corresponding author upon reasonable request.
